# Reply to the comment on the article: Reduction of the acquisition time needed to obtain somatosensory evoked potentials by estimation of the required averaging sweep count by an algorithm

**DOI:** 10.1007/s10877-025-01368-x

**Published:** 2025-10-09

**Authors:** Clemens Bothe

**Affiliations:** Departement of Anesthesiology , University Hospital Schleswig-Holstein , Kiel, Germany

Thank you for your interest in our study and your thoughtful supplements and comments concerning the methodology of somatosensory evoked potentials (SEP) [1].

As you mentioned, it has to be emphasized that it is crucial to be able to adapt to the everchanging environment of patient status during surgery. Hence, the idea of the study and the proposed algorithm was not intended to render the sweep counts as low as possible for every patient, thereby disregarding the patient specific physiological changes and the specific surgical procedure, but to investigate, if it is possibe to identify patients that would allow for safe reduction of sweep count in principle.

Hence, we discussed in the paper, that the algorithm will need to be further evaluated in patients with preexisting neurological diseases and comorbidities, as well as during specific sugeries. The study only showed that it is possible to reduce the sweep counts in order to speed up the process during stable conditions, in principle. Moreover, no clinical application of medical procedures should be derived from one study alone. Further confirmation, algorithm refinement, machine language programming and clinical evaluation are part of our ongoing research work.

We are also grateful for emphasizing the importance of morphological changes in waveforms. We will evaluate the curves for deformation of SEP morphology during reduction of the sweep count as a hint to SEP morphology deformation due to sweep count reduction and include waveform analysis in our further algorithm evalution and refinement.

Finally, no algorithm does substitute careful evaluation of the SEP by the neurophysiologist or anesthesiologist.

## Data Availability

No datasets were generated or analysed during the current study.
